# Racial/Ethnic, Nativity, and Sociodemographic Disparities in Maternal Hypertension in the United States, 2014-2015

**DOI:** 10.1155/2018/7897189

**Published:** 2018-05-17

**Authors:** Gopal K. Singh, Mohammad Siahpush, Lihua Liu, Michelle Allender

**Affiliations:** ^1^Office of Health Equity, Health Resources and Services Administration, US Department of Health and Human Services, 5600 Fishers Lane, Rockville, MD 20857, USA; ^2^Department of Health Promotion, College of Public Health, University of Nebraska Medical Center, Omaha, NE 68198, USA; ^3^Department of Preventive Medicine, Keck School of Medicine, University of Southern California, Los Angeles, CA 90032, USA

## Abstract

This study examines racial/ethnic, nativity, and sociodemographic variations in the prevalence of maternal hypertension in the United States. The 2014-2015 national birth cohort data (*N* = 7,966,573) were modeled by logistic regression to derive unadjusted and adjusted differentials in maternal hypertension consisting of both pregnancy-related hypertension and chronic hypertension. Substantial racial/ethnic differences existed, with prevalence of maternal hypertension ranging from 2.2% for Chinese and 2.9% for Vietnamese women to 8.9% for American Indians/Alaska Natives (AIANs) and 9.8% for non-Hispanic blacks. Compared with Chinese women, women in all other ethnic groups had significantly higher risks of maternal hypertension, with Filipinos, non-Hispanic blacks, and AIANs showing 2.0 to 2.9 times higher adjusted odds. Immigrant women in most racial/ethnic groups had lower rates of maternal hypertension than the US-born, with prevalence ranging from 1.9% for Chinese immigrants to 10.3% for US-born blacks. Increasing maternal age, lower education, US-born status, nonmetropolitan residence, prepregnancy obesity, excess weight gain during pregnancy, and gestational diabetes were other important risk factors. AIANs, non-Hispanic whites, blacks, Puerto Ricans, and some Asian/Pacific Islander subgroups were at substantially higher risk of maternal hypertension. Ethnicity, nativity status, older maternal age, and prepregnancy obesity and excess weight gain should be included among the criteria used for screening for gestational hypertension.

## 1. Introduction

Hypertension in pregnancy is associated with an increased risk for a number of pregnancy complications and adverse birth outcomes [1–3]. Pregnancy-related hypertension is one of the leading causes of maternal mortality in the United States [4]. Indeed, pregnancy-related hypertension, along with abortion and hemorrhage, accounts for approximately 50% of all maternal deaths worldwide [5]. Women with hypertension in pregnancy have a greater risk of developing hypertension, stroke, cardiovascular disease, and type 2 diabetes later in life than those without gestational hypertension [1, 6–8]. Women with gestational hypertension also have a significantly higher risk of dysfunctional and prolonged labor, induced labor, placental abruption, cesarean section, postpartum depressive symptoms, and poor health status [1, 6–8]. Gestational hypertension and preeclampsia are also important risk factors for neonatal morbidity and mortality [1, 6–8]. Preeclampsia is associated with an increased risk of preterm birth, small for gestational age, and low birthweight [1, 6–8]. Children born to hypertensive mothers have been shown to have higher rates of admission to neonatal intensive care units, resulting in higher healthcare costs [7, 8]. Children of mothers with gestational hypertension are themselves at increased risk of elevated blood pressure during adolescence [7].

Data from the National Vital Statistics System indicate a steady rise in the prevalence of pregnancy-related hypertension in the United States, from 2.9% in 1989 to 5.6% in 2015 [9]. Prevalence of maternal hypertension consisting of both chronic and pregnancy-related hypertension is more than doubled from 3.5% in 1989 to 7.2% in 2015 [9].

In spite of many known adverse health effects of hypertension in pregnancy, racial/ethnic, sociodemographic, and medical risk factors associated with increased risk of maternal hypertension have not been well studied in the United States. Although several studies have documented broad racial/ethnic variations in maternal hypertension, few studies have analyzed variations across a wide range of racial/ethnic and immigrant groups in the US [6, 8, 10–13]. Specifically, the prevalence of maternal hypertension for specific Asian/Pacific Islander (API) and Hispanic subgroups is not known. In addition, although such characteristics as maternal age, smoking, marital status, maternal education, gestational diabetes, prepregnancy body mass index (BMI), and weight gain during pregnancy have been mentioned as possible risk factors for maternal hypertension, few studies have examined the role of these factors simultaneously [7, 12, 13]. A better understanding of maternal hypertension risks and their determinants among major racial/ethnic and immigrant groups is vital to improve maternal health and health outcomes among mothers and children in the US.

The primary aim of this study was to examine the extent of racial/ethnic variation in the prevalence of maternal hypertension in the United States and to identify relevant sociodemographic and medical risk factors, using national data. The study also examines whether racial/ethnic variation in maternal hypertension varies according to nativity/immigrant status. Since immigration is a major characteristic of the Asian and Hispanic populations and nearly a quarter of all US births occur among foreign-born mothers [9, 14], our analysis is stratified by nativity status to highlight immigrant differences in maternal hypertension within each racial/ethnic group.

Maternal hypertension, a checkbox item as a medical risk factor on the birth certificate, is defined as blood pressure exceeding 140/90 mmHg during pregnancy [15]. Maternal hypertension includes both chronic (preexisting) hypertension as well as gestational or pregnancy-related hypertension [10, 15]. We also consider disparities in chronic hypertension, pregnancy-related hypertension and eclampsia separately, although distinguishing different types of hypertensive disorders remains a challenge on the birth certificates [15]. Eclampsia, a serious medical condition, is the final stage of preeclampsia that causes seizures/convulsions usually late in the pregnancy [16].

## 2. Methods

The maternal hypertension data in this study are derived from the birth certificates filed in the 50 US states, the District of Columbia, and New York City [9, 15]. These data, included in the annual national natality files, have been collected on the birth certificates since 1989 by the Centers for Disease Control and Prevention's National Center for Health Statistics [9, 15]. The birth certificate data include a wide range of maternal and infant characteristics, medical risk factors and complications, and birth outcomes, such as maternal and paternal age, race/ethnicity, nativity, marital status, education, place of residence, parity, birthweight, gestational age, prenatal care, tobacco and alcohol use during pregnancy, prepregnancy BMI, gestational weight gain, prepregnancy diabetes, gestational diabetes, hypertensive disorders in pregnancy, uterine bleeding, placenta previa, prolonged labor, and induction of labor. Information on demographic characteristics such as race/ethnicity, age, nativity, marital status, education, prepregnancy weight and height, and smoking before and during pregnancy is reported directly by the mother. However, information on obstetric procedures, characteristics of labor and delivery, and medical risk factors such as gestational diabetes and hypertension (chronic, gestational, and eclampsia) is collected directly from the medical records at the hospital or the freestanding birthing center where the birth occurs [15, 17–19]. It has to be a confirmed diagnosis of elevated blood pressure for it to be included in the patient's medical records/charts. Obstetricians/gynecologists, physician assistants, or nurse practitioners are generally the healthcare providers who make the medical risk factor diagnoses during pregnancy [17, 19]. Detailed descriptions of the birth certificate data and national natality files are available elsewhere [9, 15].

We used the 2014 and 2015 national birth cohort data [9, 15]. During 2014-2015, 7,966,573 births occurred among US mothers. For all births, information on whether or not mothers had pregnancy-related or chronic hypertension was available [15]. Of 7,966,573 women who gave birth during 2014-2015, 424,704 had pregnancy-related hypertension, 128,267 had chronic hypertension, and 19,278 mothers had eclampsia. In all, 552,971 mothers were diagnosed with hypertension in pregnancy during 2014-2015. Aggregating data for two years ensured sufficient sample sizes for analyzing hypertension patterns among groups stratified by race/ethnicity and immigrant status.

Race/ethnicity was classified into 17 major categories: Non-Hispanic whites, Non-Hispanic blacks, AIANs, Chinese, Asian Indians, Filipinos, Japanese, Koreans, Vietnamese, Hawaiians, Samoans, and other Asian/Pacific Islanders, Mexicans, Puerto Ricans, Cubans, Central and South Americans, and other Hispanics. Immigrant status was defined on the basis of mothers' place of birth [9, 11, 15]. US-born categories were those born in one of the 50 states or Washington, DC. Immigrants or foreign-born categories refer to those born outside these geographic areas [9, 11, 15]. The joint variable of ethnic-immigrant status included 31 categories, with each racial/ethnic group divided into the US-born and foreign-born categories. Note that although AIANs, Hawaiians, and Samoans are considered native-born in the present analysis, a small percentage of AIANs and Hawaiians and 30% of Samoans are born outside the 50 states and Washington, DC [9, 11].

In addition to race/ethnicity and immigrant status, we considered the following sociodemographic and medical risk factors associated with maternal hypertension that were available in the natality files: maternal age, marital status, maternal education, metropolitan/nonmetropolitan residence, geographic region of residence, gestational diabetes, prepregnancy BMI, gestational weight gain, and smoking before and during pregnancy [6–8, 12, 13]. All covariates except smoking were measured as shown in Tables 1 and 2. Smoking before and during pregnancy was defined as dichotomous variables with “yes” and “no” categories.

Prevalence estimates and prevalence ratios were used to describe the overall association between covariates and maternal hypertension. Prevalence ratio was defined as the ratio of the prevalence for a specific group to that for the reference group. Multivariable logistic regression was used to model the adjusted association between each sociodemographic characteristic and the risk of maternal hypertension, pregnancy-related hypertension, chronic hypertension, or eclampsia [20]. In estimating the odds of hypertension for race/ethnicity and ethnic-immigrant status, we considered Chinese women or Chinese immigrant women as the reference group based on prior research and because they had the lowest prevalence, which could potentially be achievable by other population subgroups [11, 21, 22]. Fitted logistic models were used to derive adjusted hypertension prevalence at mean values of the covariates [20, 21].

An index of disparity, which approximated in relative terms the average deviation of the rates from the rate for the best-off racial/ethnic or ethnic-nativity group, was used to summarize hypertension disparities across all groups [23, 24]. This relative mean deviation index of disparity was calculated as

(1) $$\begin{array}{c} ID=\left\{\;\frac{\left(\sum_{i}\left|H_{ri}-H_{rl}\right|/I\right)}{H_{rl}}\;\right\}\times 100;\;H_{rl}>0, \end{array}$$

where *H*_*ri*_ is the hypertension prevalence for the *i*th group (*i* = 1,2, 3,…, 31), *H*_*rl*_ is the prevalence for the group with the lowest prevalence (i.e., Chinese women), and *I* is the number of racial/ethnic (17) or ethnic-immigrant groups (31) being compared [20].

## 3. Results

During 2014-2015, the overall prevalence of maternal hypertension in the US was 6.9%. About 5.3% of women had pregnancy-related hypertension and 1.6% had chronic hypertension. Substantial racial/ethnic differences existed in the prevalence of maternal hypertension, ranging from 2.2% for Chinese and 2.9% for Vietnamese women to 8.9% for American Indians/Alaska Natives (AIANs) and 9.8% for non-Hispanic blacks (Table 1). Compared to non-Hispanic white women, other Asian groups such as Japanese, Koreans, and Asian Indians had significantly lower prevalence, while Filipinos, Samoans, AIANs, and non-Hispanic blacks had significantly higher prevalence. Compared to Chinese women, women in all other racial/ethnic groups had significantly higher risks of maternal hypertension. Among API women, Samoans, Filipinos, and Hawaiians had the highest prevalence of maternal hypertension. Among Hispanics, Puerto Ricans had the highest prevalence (6.4%), followed by Cubans, Mexicans, and Central and South Americans. However, the prevalence for all Hispanic subgroups was significantly lower than the prevalence of 7.2% for non-Hispanic whites (Table 1).

Immigrant women in most racial/ethnic groups had lower rates of maternal hypertension than their US-born counterparts, with the prevalence ranging from 1.9% for Chinese immigrants and 2.3% for Japanese immigrants to 10.3% for US-born blacks (Table 2). For example, Chinese immigrants had a 56% lower risk of maternal hypertension than US-born Chinese, Vietnamese immigrants had a 44% lower risk than US-born Vietnamese, black immigrants had a 31% lower risk than US-born blacks, Mexican immigrants had a 22% lower risk than US-born Mexicans, and non-Hispanic white immigrants had a 45% lower risk than US-born non-Hispanic whites.

Racial/ethnic groups varied greatly in their sociodemographic and medical characteristics known to be associated with maternal hypertension (Table 3). For example, while <13% of births occurred among AIAN, Puerto Rican, and black mothers aged ≥35 years, this percentage was 32% among Chinese and Filipinos, 37% among Koreans, and 49% among Japanese mothers. Educational attainment was the highest among Asian Indian and Korean women and the lowest among Mexican and Samoan women. The percentage of mothers with a college degree ranged from 76.7% for Koreans and 78.3% for Asian Indians to 7.4% for Samoans, 9.1% for Mexicans, and 9.2% for AIANs. More than 87% of Chinese and Asian Indian mothers were foreign-born, compared with 6.6% of non-Hispanic whites and 15.0% of blacks. The prevalence of gestational diabetes was 13.3% for Asian Indians, 11.9% for Filipinos, 11.6% for Vietnamese, 10.1% for Samoans, 10% for AIANs, and 9.5% for Chinese, compared with 5.6% for blacks and 5.7% for non-Hispanic whites. Samoan women had the highest prevalence of prepregnancy obesity (64.4%), followed by Hawaiians (37.2%), AIANs (36.0%), blacks (35.0%), Puerto Ricans (29.9%), and Mexicans (29.1%). Chinese women had the lowest prepregnancy obesity prevalence (2.7%). Samoans, Hawaiians, Cubans, Puerto Ricans, non-Hispanic Whites, Blacks, and AIANs were significantly more likely to experience excess weight gain (>40 pounds) during pregnancy compared to women in all Asian subgroups. Approximately 22.3% of AIANs and 14.9% of non-Hispanic whites reported having smoked before pregnancy, compared with <3% of Asian mothers. Racial/ethnic patterns in smoking during pregnancy were similar.

After controlling for covariates, women in all racial/ethnic groups had significantly higher risks of maternal hypertension compared to Chinese women (Table 1, Model 2). Compared with Chinese women, non-Hispanic whites, AIANs, non-Hispanic blacks, and Filipinos had, respectively, 1.9, 2.0, 2.4, and 2.9 times higher adjusted odds of maternal hypertension. Compared with Chinese women, Mexicans, Central and South Americans, Puerto Ricans, and Cubans had, respectively, 1.5, 1.6, 1.7, and 1.8 times higher adjusted odds of maternal hypertension. Compared with Chinese immigrants, the adjusted odds of maternal hypertension were 2.1 times higher for US-born Chinese, 2.6 times higher for black immigrants, 3.5 times higher for US-born blacks, 1.7 times higher for non-Hispanic whites, 2.8 times higher for US-born non-Hispanic whites, 3.5 times higher for Filipino immigrants, 3.4 times higher for US-born Filipinos, 3.0 times higher for US-born Japanese, and 2.9 times higher for AIANs (Table 2, Model 2). Sociodemographic and medical risk factors accounted for 63% of racial/ethnic disparities and 46% of ethnic-immigrant disparities in maternal hypertension, based on comparison of the disparity indices for the unadjusted and adjusted prevalence estimates (data not shown).

Table 1 shows variation in the prevalence and odds of maternal hypertension according to other sociodemographic characteristics. Increasing maternal age, unmarried status, US-born status, lower education, nonmetropolitan residence, residence in the Southern United States, prepregnancy obesity, excess weight gain during pregnancy, and gestational diabetes were all associated with increased risks of maternal hypertension. Women aged 40–44 and ≥45 years had, respectively, 1.7 and 2.5 times higher adjusted odds of maternal hypertension than those aged <20 years. Women with gestational diabetes had a 2.7 times higher prevalence and 2.4 times higher adjusted odds of maternal hypertension than those who did not have gestational diabetes. Compared to women with normal weight (BMI < 25), overweight women as well as women with grade 1 and grade 2 obesity had, respectively, 1.7, 2.7, and 4.6 times higher adjusted odds of maternal hypertension. Women who gained >40 pounds had 94% higher adjusted odds of maternal hypertension than those who gained <16 pounds during pregnancy. Women in the Southeastern and Southwestern United States had 33–35% higher adjusted odds of maternal hypertension than those living in the Pacific region. Although smoking during and before pregnancy was associated with 10% and 18% higher odds of maternal hypertension, respectively, after controlling for sociodemographic and medical risk factors, smoking was found to be not significantly related to maternal hypertension (data not shown).

Racial/ethnic and ethnic-immigrant disparities in pregnancy-related hypertension and chronic hypertension, presented in Tables 4 and 5, respectively, generally show patterns similar to those for the combined outcome of maternal hypertension. However, the effect-sizes for some risk factors such as maternal age, prepregnancy BMI, smoking during and before pregnancy, education, and geographic residence were stronger for chronic hypertension than for pregnancy-related hypertension (data not shown for brevity). For example, compared to those aged <20 years, women aged 40–44 and ≥45 years had, respectively, 8.8 and 12.0 times higher adjusted odds of chronic hypertension and 1.1 and 1.6 times higher adjusted odds of pregnancy-related hypertension. Grade 1 and grade 2 prepregnancy obesity were associated with 3.7 and 7.8 times higher adjusted odds of chronic hypertension and 2.5 and 3.7 times higher adjusted odds of pregnancy-related hypertension. While maternal education was not significantly related to pregnancy-related hypertension after controlling for other covariates, there was a consistent and inverse educational gradient in chronic hypertension. Women with less than a high school education had 30% higher adjusted odds of chronic hypertension than those with a college degree. Women in the Southeastern United States had 87% higher adjusted odds and those in the New England region had 62% higher adjusted odds of chronic hypertension than those living in the Pacific region. While smoking was not significantly related to pregnancy-related hypertension, smoking before and during pregnancy was associated with 30–33% higher risks of chronic hypertension. Smoking before or during pregnancy was associated with 58-59% higher age-adjusted odds of chronic hypertension and 17-18% higher covariate-adjusted odds of chronic hypertension.

**Figure 1:**
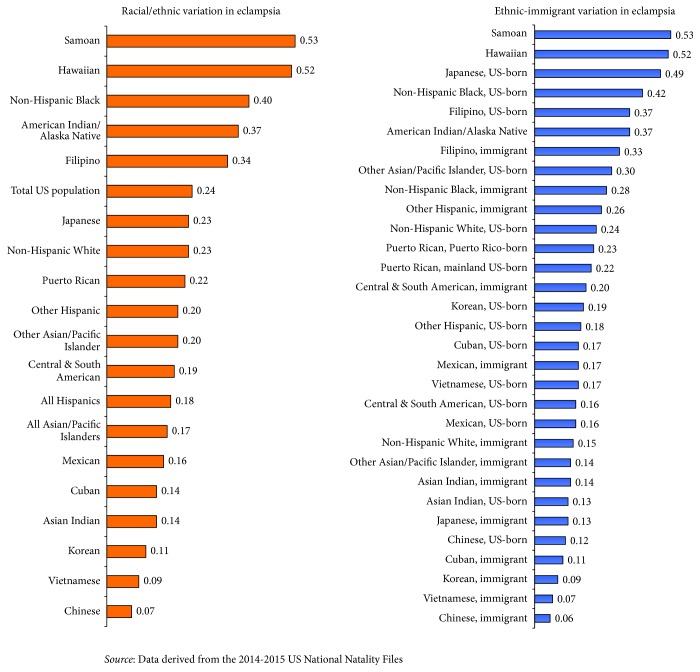
Prevalence (%) of eclampsia among women in 17 racial/ethnic and 31 ethnic-immigrant groups, United States, 2014-2015.

Racial/ethnic variations in eclampsia were similar to those for chronic and pregnancy-related hypertension. Prevalence of eclampsia was the highest among Samoan, Hawaiian, Non-Hispanic black, AIAN, and Filipino women and the lowest among Chinese, Vietnamese, and Korean women (Figure 1). Compared with Chinese women, blacks, Japanese, Samoans, Filipinos, and Hawaiians had 3 to 4 times higher adjusted odds of eclampsia (data not shown). Immigrant women in most racial/ethnic groups had a lower risk of eclampsia than their US-born counterparts. The rate of eclampsia ranged from 0.6 per 1,000 live births for Chinese immigrant women to 5.2 for Hawaiians and 5.3 per 1,000 live births for Samoans (Figure 1). Chinese immigrants had 47% lower adjusted odds than US-born Chinese; Japanese immigrants had 72% lower adjusted odds than US-born Japanese; black immigrants had 17% lower adjusted odds than US-born blacks; and white immigrants had 19% lower adjusted odds of eclampsia than US-born whites (data not shown). Maternal age < 20 and ≥45 years was associated with substantially increased risks of eclampsia. Women in the Southeast region had 2.3 times higher adjusted odds of eclampsia than those in the Pacific region. Gestational diabetes, grade 1 prepregnancy obesity, and grade 2 prepregnancy obesity were associated with 2.0, 2.0, and 2.7 times higher adjusted odds of eclampsia, respectively (data not shown).

## 4. Discussion

To our knowledge, this is the largest population-based study of maternal hypertension in the United States. The results of this national study indicate substantial racial/ethnic and nativity differences in the risk of maternal hypertension, which were only partially explained by differences in maternal age, education, prepregnancy BMI, weight gain during pregnancy, gestational diabetes, and other relevant sociodemographic characteristics. The detailed analysis of maternal hypertension prevalence among specific API and Hispanic subgroups as well as among a large number of immigrant groups is a particularly novel feature of our study. The increased risks of maternal hypertension among AIANs, non-Hispanic whites, blacks, and Puerto Ricans are consistent with those reported in previous US studies [6, 8, 10–12]. Significantly higher risks of maternal hypertension among Filipinos, Samoans, and Hawaiians and lower risks among other Asian groups such as Chinese, Japanese, Koreans, and Vietnamese reported in our study are consistent with two US studies conducted in Hawaii and New York City that show similar racial/ethnic patterns in maternal hypertension and preeclampsia [8, 13]. Our findings of lower risks of maternal hypertension among Mexicans, Cubans, and Central/South Americans are compatible with previous studies that show lower risks of hypertension among Hispanics compared to non-Hispanic whites but higher risks among Hispanics when compared to Asian groups [4, 8, 11, 12].

Racial/ethnic patterns in maternal hypertension documented here are largely consistent with those observed in hypertension among the adult population and for reproductive age women in the US [25, 26]. Data from the 2010–2014 US National Health Interview Survey show that non-Hispanic black women aged 18–49 had the highest prevalence of hypertension (22.3%), followed by Native Hawaiians/Pacific Islanders (20.0%), AIANs (16.0%), Filipinos (12.8%), and non-Hispanic whites (12.2%). Chinese, Asian Indians, and other Asians including Japanese, Korean, and Vietnamese women aged 18–49 had the lowest prevalence (<7%) [26].

Besides race/ethnicity and immigrant status, the other important predictors of maternal hypertension included maternal age, gestational diabetes, prepregnancy BMI, and gestational weight gain, which are consistent with previous studies [7, 8, 12, 13]. The finding of higher prevalence of maternal hypertension in the Midwest and Southern regions is consistent with the previously reported regional patterns in adult hypertension [25]. Increased risk of maternal hypertension and chronic hypertension associated with lower education is in line with the previously reported positive relationship between low socioeconomic status (SES) and gestational hypertension [8, 27, 28]. The finding that immigrants in most racial/ethnic groups have a lower risk of maternal hypertension than their US-born counterparts is consistent with studies that show significantly lower rates of maternal hypertension and adult hypertension among immigrants [11, 13, 29].

Although immigrants account for 13.5% of the total US population, immigrant women make up 20.4% of the reproductive-age population [14]. Given the marked inequalities in maternal hypertension by immigrant status, the magnitude of health disparities is likely to be substantial for women in reproductive ages [11, 17]. Although immigrant women in most racial/ethnic groups had lower rates of maternal hypertension than the US-born, their reduced risks of hypertension and other health advantages are likely to diminish with increasing acculturation levels or duration of residence in the US [11, 17]. Although genetic factors might partly explain racial/ethnic disparities in maternal hypertension, lower risks among immigrants relative to native-born women of similar ethnicities indicate the significance of social environments, acculturation, and lifestyle factors [11, 17]. Ethnic-minority and socially disadvantaged groups in the US differ greatly from the majority, affluent groups in their social, physical, and living environments that might influence hypertension, and related risks such as prepregnancy obesity, gestational diabetes, and weight gain during pregnancy. They have limited access to neighborhood amenities such as sidewalks, parks/playgrounds, green spaces, public transportation, and healthy, affordable foods that promote physical activity, healthy lifestyle, and healthy living [11, 21, 22, 30].

Our study has limitations. Because of lack of data, other important risk factors for maternal hypertension such as diet, physical activity, family history of hypertension, and the social and built environments, which could explain some of the reported racial/ethnic and nativity differences, could not be taken into account. Socioeconomic data in our study were also limited, with maternal education being the only SES measure available. Other measures of SES such as family income, occupation, and neighborhood deprivation, which have been associated with gestational hypertension and adult hypertension, were lacking in our database [7, 15, 31]. Moreover, because of the nature and quality of the birth certificate data, we could not fully distinguish between different hypertensive disorders of pregnancy such as chronic hypertension, preeclampsia, eclampsia, preeclampsia superimposed on chronic hypertension, and gestational hypertension [15]. Some of the women who did not receive timely and regular prenatal care in our study might have been missed from being screened for gestational hypertension. Studies have found underreporting of hypertensive disorders including chronic hypertension, pregnancy-induced hypertension, and preeclampsia on birth certificates when compared with hospital discharge data [32–34]. Underreporting of hypertension is found across all racial/ethnic and socioeconomic groups [32]. However, the degree of underreporting is noted to be higher among women with lower education and income levels, which may have affected the socioeconomic gradients in the overall outcome of maternal hypertension and specific hypertensive disorders shown here [32]. The major strength of our national study is its large sample size of 8 million women, which allowed us to compare risks of maternal hypertension and related risk factors among a large number of racial/ethnic and immigrant groups. Such subgroup comparisons were not feasible in previously smaller studies.

## 5. Conclusions

This large population-based study of 8 million US women has shown considerable heterogeneity in maternal hypertension prevalence across various racial/ethnic and immigrant groups. Non-Hispanic whites and several ethnic-minority groups such as non-Hispanic blacks, AIANs, Samoans, Hawaiians, Filipinos, and Puerto Ricans have relatively high levels of maternal hypertension, exceeding 6%. Most Asian groups including Chinese, Japanese, Vietnamese, Koreans, and Asian Indians have substantially lower prevalence of maternal hypertension (<4%). High rates of maternal hypertension correspond closely with the higher prevalence of important risk factors and perinatal outcomes among these groups such as prepregnancy obesity, excess weight gain during pregnancy, smoking before and during pregnancy, lower maternal education, pregnancy complications, preterm birth, and neonatal mortality [4, 9–11]. For most racial/ethnic groups, immigrants have substantially lower rates of maternal hypertension than their US-born counterparts. These findings highlight the significance of stratifying analyses by immigrant status and suggest ethnic-specific and culturally appropriate interventions to prevent and control hypertension and related risks such as prepregnancy obesity among women of reproductive age and to improve health outcomes [11, 17]. Tackling the rising prevalence of chronic hypertension, gestational hypertension, obesity, preexisting and gestational diabetes, and cardiovascular conditions among women of reproductive age should become a priority if we are to further improve maternal health and reduce health disparities in the United States [35]. Further research is needed to assess the role of social, behavioral, and environmental factors responsible for ethnic, immigrant, and sociodemographic disparities in maternal hypertension.

## Figures and Tables

**Table 1:** Observed prevalence and multivariate logistic regressions showing age- and covariate-adjusted differentials in *maternal hypertension* among major racial/ethnic groups and by selected social and medical characteristics, United States, 2014-2015 (*N* = 7,966,573).

Covariate	Number of births	Prevalence percent	Prevalence ratio^1^	Model 1^2^			Model 2^3^			Covariance-adjusted	
				OR	95% CI		OR	95% CI		prevalence	SE
*Race/ethnicity*											
Non-Hispanic White	4,322,169	7.17	3.32^*∗*^	3.86	3.71	4.02	1.92	1.84	2.00	7.05	0.01
Non-Hispanic Black	1,188,014	9.82	4.55^*∗*^	5.66	5.44	5.89	2.42	2.32	2.52	8.62	0.03
American Indian/Alaska Native	75,204	8.93	4.13^*∗*^	5.19	4.95	5.44	2.00	1.91	2.10	7.32	0.09
Chinese	116,439	2.16	1.00	1.00	Reference		1.00	Reference		3.92	0.08
Japanese	14,177	3.45	1.60^*∗*^	1.49	1.35	1.64	1.50	1.36	1.66	5.66	0.24
Filipino	63,160	7.74	3.58^*∗*^	3.83	3.65	4.03	2.94	2.79	3.08	10.18	0.13
Hawaiian	1,725	6.96	3.22^*∗*^	3.80	3.14	4.59	1.58	1.30	1.91	5.92	0.51
Asian Indian	131,594	3.98	1.84^*∗*^	2.00	1.90	2.10	1.55	1.48	1.63	5.85	0.08
Korean	30,390	3.60	1.67^*∗*^	1.65	1.54	1.77	1.52	1.42	1.64	5.75	0.16
Vietnamese	41,211	2.93	1.36^*∗*^	1.38	1.29	1.48	1.28	1.19	1.37	4.90	0.13
Samoan	4,316	8.60	3.98^*∗*^	4.89	4.37	5.48	1.62	1.44	1.81	6.05	0.30
Other Asian/Pacific Islander	140,061	4.45	2.06^*∗*^	2.28	2.17	2.39	1.55	1.47	1.62	5.82	0.07
Mexican	1,092,146	5.16	2.39^*∗*^	2.79	2.68	2.91	1.48	1.42	1.54	5.60	0.03
Puerto Rican	140,866	6.35	2.94^*∗*^	3.55	3.39	3.71	1.70	1.62	1.78	6.34	0.06
Cuban	41,270	5.86	2.71^*∗*^	3.10	2.93	3.29	1.82	1.72	1.93	6.73	0.13
Central & South American	278,905	4.54	2.10^*∗*^	2.33	2.23	2.43	1.61	1.54	1.68	6.03	0.05
Other Hispanic	284,926	6.00	2.78^*∗*^	3.32	3.18	3.47	1.69	1.61	1.76	6.29	0.05
*Maternal age (years)*											
<20	484,062	6.30	1.00	1.00	Reference		1.00	Reference		6.25	0.04
20–24	1,733,076	6.09	0.97^*∗*^	0.97	0.95	0.98	0.82	0.81	0.83	5.43	0.02
25–29	2,297,703	6.48	1.03^*∗*^	1.03	1.02	1.04	0.88	0.86	0.89	5.95	0.02
30–34	2,175,751	6.99	1.11^*∗*^	1.12	1.10	1.13	0.99	0.97	1.00	6.53	0.02
35–39	1,036,744	8.50	1.35^*∗*^	1.38	1.36	1.40	1.21	1.19	1.23	7.81	0.03
40–44	221,869	11.34	1.80^*∗*^	1.90	1.87	1.94	1.67	1.64	1.70	10.02	0.06
≥45	17,368	16.09	2.55^*∗*^	2.86	2.74	2.98	2.49	2.38	2.61	14.00	0.27
*Marital status*											
Married	4,760,176	6.67	1.00	1.00	Reference		1.00	Reference		6.20	0.01
Unmarried	3,206,397	7.34	1.10^*∗*^	1.25	1.24	1.26	1.07	1.07	1.08	6.55	0.02
*Nativity/immigrant status*											
US born	6,176,861	7.61	1.65^*∗*^	1.84	1.83	1.86	1.29	1.28	1.30	6.63	0.01
Foreign born	1,771,387	4.60	1.00	1.00	Reference		1.00	Reference		5.10	0.02
*Maternal education (years)*											
<12	1,134,548	5.94	0.91^*∗*^	1.02	1.01	1.03	0.92	0.91	0.93	6.59	0.03
12	1,925,949	7.03	1.08^*∗*^	1.25	1.24	1.26	0.98	0.97	0.99	7.08	0.02
13–15	2,257,033	8.09	1.24^*∗*^	1.41	1.40	1.42	1.06	1.06	1.07	7.61	0.02
≥16	2,343,837	6.50	1.00	1.00	Reference		1.00	Reference		7.13	0.02
*Plurality*											
Single	7,689,433	6.70	1.00	1.00	Reference		1.00	Reference		6.16	0.01
Multiple	277,140	13.73	2.05^*∗*^	2.12	2.09	2.14	1.82	1.80	1.84	10.66	0.06
*Gestational diabetes*											
No	7,446,975	6.26	1.00	1.00	Reference		1.00	Reference		5.69	0.01
Yes	508,788	17.09	2.87^*∗*^	2.95	2.93	2.97	2.44	2.42	2.46	13.24	0.05
*Pre-pregnancy BMI*											
Normat weight (BMI < 25)	3,679,645	3.95	1.00	1.00	Reference		1.00	Reference		3.54	0.01
Overweight (25 ≤ BMI < 30)	1,923,934	6.86	1.74^*∗*^	1.78	1.77	1.80	1.74	1.73	1.76	5.83	0.02
Obesity grade 1 (30 ≤ BMI < 35)	1,034,214	10.26	2.60^*∗*^	2.77	2.75	2.80	2.69	2.67	2.71	8.45	0.03
Obesity grade 2 (BMI ≥ 35)	846,413	16.74	4.24^*∗*^	4.91	4.87	4.95	4.60	4.56	4.64	11.22	0.05
*Weight gain during pregnancy (lbs)*											
<16	1,166,852	7.96	1.00	1.00	Reference		1.00	Reference		5.41	0.02
16–30	2,938,813	5.88	0.74^*∗*^	1.10	1.09	1.10	1.12	1.11	1.13	5.77	0.01
31–40	1,899,825	6.05	0.76^*∗*^	1.32	1.30	1.33	1.32	1.31	1.34	6.57	0.02
>40	1,624,798	9.16	1.15^*∗*^	2.04	2.02	2.06	1.94	1.92	1.95	9.25	0.02
*Place of residence*											
Metropolitan county	6,353,967	6.68	1.00	1.00	Reference		1.00	Reference		6.27	0.01
Non-metropolitan county	1,612,606	7.98	1.19^*∗*^	1.27	1.26	1.28	1.04	1.04	1.05	6.57	0.02
*Region of residence*											
New England	299,320	6.12	1.15^*∗*^	1.15	1.13	1.17	1.10	1.08	1.11	5.55	0.04
Mid Atlantic	965,794	6.25	1.18^*∗*^	1.19	1.18	1.20	1.10	1.09	1.11	5.24	0.02
East Northcentral	1,125,412	7.96	1.50^*∗*^	1.60	1.58	1.62	1.24	1.23	1.26	6.69	0.02
West Northcentral	548,470	7.52	1.42^*∗*^	1.51	1.49	1.53	1.19	1.17	1.21	6.36	0.03
South Atlantic	1,499,665	7.16	1.35^*∗*^	1.42	1.40	1.43	1.15	1.14	1.16	6.40	0.02
East Southcentral	471,637	8.87	1.67^*∗*^	1.85	1.83	1.88	1.35	1.33	1.37	7.77	0.04
West Southcentral	1,116,431	7.51	1.42^*∗*^	1.52	1.51	1.54	1.33	1.32	1.35	7.30	0.03
Mountain	616,787	6.53	1.23^*∗*^	1.29	1.28	1.31	1.19	1.17	1.20	6.49	0.03
Pacific	1,323,057	5.30	1.00	1.00	Reference		1.00	Reference		5.50	0.02

OR = odds ratio; CI = confidence interval. ^*∗*^Statistically significant at *p* < 0.05. ^1^Defined as the ratio of the prevalence for a specific group to that for the reference group. ^2^Adjusted for maternal age only; weight gain odds were adjusted for maternal age and prepregnancy BMI. ^3^Adjusted for maternal age, marital status, nativity, plurality, maternal education, place and region of residence, gestational diabetes, prepregnancy BMI, and gestational weight gain. *Source*. Data derived from the 2014-2015 US National Natality data files.

**Table 2:** Observed prevalence and adjusted odds of *maternal hypertension* among 32 ethnic-immigrant groups: United States, 2014-2015 (*N* = 7,966,573).

Ethnic-immigrant group	Number of births	Prevalence percent	Prevalence ratio^1^	Model 1^2^			Model 2^3^			Covariance-adjusted	
				OR	95% CI		OR	95% CI		prevalence	SE
Non-Hispanic White, US-born	4,031,903	7.39	3.97^*∗*^	4.73	4.52	4.95	2.77	2.65	2.90	7.41	0.01
Non-Hispanic White, immigrant	282,556	4.08	2.19^*∗*^	2.32	2.21	2.43	1.73	1.65	1.82	4.85	0.04
Non-Hispanic Black, US-born	1,004,997	10.30	5.54^*∗*^	7.27	6.94	7.61	3.47	3.31	3.64	9.01	0.03
Non-Hispanic Black, immigrant	177,299	7.08	3.81^*∗*^	4.13	3.93	4.34	2.63	2.50	2.76	7.07	0.06
American Indian/Alaska Native	75,204	8.93	4.80^*∗*^	6.19	5.87	6.52	2.86	2.71	3.01	7.61	0.09
Chinese, US-born	14,497	4.25	2.28^*∗*^	2.31	2.10	2.53	2.12	1.93	2.33	5.83	0.22
Chinese, immigrant	101,753	1.86	1.00	1.00	Reference		1.00	Reference		2.93	0.06
Japanese, US-born	3,893	6.45	3.47^*∗*^	3.43	3.00	3.93	3.02	2.63	3.46	7.98	0.46
Japanese, immigrant	10,270	2.32	1.25^*∗*^	1.12	0.98	1.28	1.28	1.11	1.46	3.68	0.23
Hawaiian	1,725	6.96	3.74^*∗*^	4.49	3.71	5.43	2.23	1.84	2.71	6.11	0.53
Filipino, US-born	17,216	7.99	4.30^*∗*^	4.83	4.49	5.18	3.43	3.19	3.69	8.91	0.22
Filipino, immigrant	45,845	7.63	4.10^*∗*^	4.33	4.09	4.58	3.53	3.33	3.73	9.13	0.14
Asian Indian, US-born	13,323	4.52	2.43^*∗*^	2.57	2.34	2.82	1.96	1.79	2.16	5.45	0.21
Asian Indian, immigrant	118,026	3.91	2.10^*∗*^	2.31	2.18	2.43	1.75	1.65	1.84	4.90	0.07
Korean, US-born	6,296	3.81	2.05^*∗*^	2.11	1.84	2.42	1.89	1.65	2.17	5.26	0.32
Korean, immigrant	23,313	3.55	1.91^*∗*^	1.87	1.72	2.03	1.78	1.64	1.94	5.00	0.16
Vietnamese, US-born	7,713	4.55	2.45^*∗*^	2.79	2.48	3.13	2.22	1.97	2.49	6.07	0.30
Vietnamese, immigrant	33,453	2.56	1.38^*∗*^	1.37	1.26	1.48	1.32	1.22	1.43	3.79	0.12
Samoan	4,316	8.60	4.62^*∗*^	5.81	5.17	6.52	2.14	1.90	2.41	5.89	0.30
Other Asian/Pacific Islander, US-born	51,424	5.11	2.75^*∗*^	3.23	3.05	3.43	2.09	1.97	2.22	5.77	0.11
Other Asian/Pacific Islander, immigrant	87,782	4.05	2.17^*∗*^	2.37	2.24	2.50	1.78	1.68	1.88	4.98	0.08
Mexican, US-born	554,774	5.79	3.12^*∗*^	3.95	3.76	4.13	2.06	1.96	2.16	5.69	0.03
Mexican, immigrant	536,471	4.51	2.43^*∗*^	2.72	2.60	2.85	1.70	1.62	1.79	4.79	0.03
Puerto Rican, mainland US-born	121,554	6.26	3.36^*∗*^	4.16	3.96	4.38	2.34	2.22	2.46	6.37	0.07
Puerto Rican, Puerto Rico-born	18,530	7.04	3.79^*∗*^	4.58	4.26	4.92	2.55	2.37	2.74	6.88	0.18
Cuban, US-born	19,864	6.16	3.31^*∗*^	3.91	3.63	4.20	2.29	2.13	2.47	6.26	0.17
Cuban, immigrant	21,389	5.59	3.00^*∗*^	3.43	3.19	3.70	2.34	2.17	2.52	6.37	0.17
Central & South American, US-born	47,914	5.30	2.85^*∗*^	3.47	3.27	3.69	2.08	1.95	2.21	5.73	0.11
Central & South American, immigrant	230,721	4.39	2.36^*∗*^	2.60	2.47	2.73	1.84	1.75	1.94	5.14	0.05
Other Hispanic, US-born	202,960	6.04	3.25^*∗*^	4.10	3.91	4.31	2.21	2.10	2.32	6.06	0.05
Other Hispanic, immigrant	81,468	5.90	3.17^*∗*^	3.61	3.42	3.81	2.41	2.28	2.54	6.54	0.09
All other ethnic-nativity groups	18,124	6.91	3.71^*∗*^	4.38	4.07	4.71	2.87	2.66	3.09	7.64	0.20

OR = odds ratio; CI = confidence interval. ^*∗*^Statistically significant at *p* < 0.05. ^1^Defined as the ratio of the prevalence for a specific group to that for the reference group. ^2^Adjusted for maternal age only. ^3^Adjusted for maternal age, marital status, plurality, maternal education, place and region of residence, gestational diabetes, prepregnancy BMI, and gestational weight gain. *Source*. Data derived from the 2014-2015 US National Natality data files.

**Table 3:** Racial/ethnic variation in selected social and medical risk factors for *maternal hypertension*, United States, 2014-2015 (*N* = 7,966,573).

Race/Ethnicity	Maternal Age ≥ 35 Years Percent	Maternal Education ≥ 16 Years Percent	Foreign Born Percent	Gestational Diabetes Percent	Pre-pregnancy Overweight (BMI ≥ 25) Prevalence (%)	Pre-pregnancy Obesity (BMI ≥ 30) Prevalence (%)	Weight gain in pregnancy (>40 lbs) Prevalence (%)	Smoking Before Pregnancy Percent	Smoking in Pregnancy Percent
All Races	16.0	30.6	22.3	6.4	50.8	25.1	21.3	10.5	8.1
Non-Hispanic White	16.3	39.6	6.6	5.7	47.3	23.1	24.0	14.9	11.6
Non-Hispanic Black	12.8	15.9	15.0	5.6	61.8	35.0	21.7	9.0	6.9
American Indian/AN	10.0	9.2	1.6	10.0	63.1	36.0	22.8	22.3	17.9
Chinese	31.6	67.5	87.5	9.5	14.3	2.7	14.7	0.4	0.2
Japanese	49.4	66.5	72.5	6.2	16.3	4.8	7.7	2.1	0.9
Filipino	31.9	53.1	72.7	11.9	36.3	11.7	16.0	2.3	1.2
Asian Indian	18.7	78.3	89.9	13.3	37.7	9.8	13.0	0.3	0.2
Korean	36.5	76.7	78.7	7.9	19.9	5.0	14.1	2.6	1.4
Vietnamese	29.2	39.9	81.3	11.6	15.8	3.6	12.9	1.0	0.6
Hawaiian	14.5	18.1	4.4	8.3	65.0	37.2	24.8	14.7	12.3
Samoan	12.0	7.4	29.6	10.1	87.6	64.4	31.2	9.9	8.1
Mexican	14.5	9.1	49.2	7.6	59.3	29.1	15.5	2.4	1.5
Puerto Rican	12.0	15.4	13.2	6.7	56.8	29.9	22.3	10.1	6.8
Cuban	17.0	26.6	51.9	5.2	47.1	19.9	25.7	3.5	2.3
Central/South American	20.8	17.5	82.8	6.4	52.0	20.5	14.9	1.2	0.6

*Source*. Data derived from the 2014-2015 US National Natality data files. AN = Alaska Native.

**Table 4:** Observed prevalence and adjusted odds of *pregnancy-related hypertension* among 17 racial/ethnic and 31 ethnic-immigrant groups, United States, 2014-2015 (*N* = 7,966,573).

Ethnicity and immigrant status	Prevalence percent	Prevalence ratio^1^	Model 1^2^			Model 2^3^		
			OR	95% CI		OR	95% CI	
*Race/ethnicity*								
Non-Hispanic White	5.67	3.22^*∗*^	3.48	3.33	3.64	1.96	1.87	2.05
Non-Hispanic Black	6.55	3.72^*∗*^	4.08	3.90	4.26	2.05	1.96	2.15
American Indian/Alaska Native	6.77	3.85^*∗*^	4.25	4.04	4.48	1.96	1.86	2.07
Chinese	1.76	1.00	1.00	Reference		1.00	Reference	
Japanese	2.69	1.53^*∗*^	1.47	1.32	1.65	1.50	1.34	1.68
Filipino	5.87	3.34^*∗*^	3.48	3.30	3.68	2.74	2.59	2.89
Hawaiian	5.62	3.19^*∗*^	3.46	2.81	4.27	1.67	1.35	2.07
Asian Indian	3.26	1.85^*∗*^	1.94	1.84	2.05	1.56	1.48	1.64
Korean	2.77	1.57^*∗*^	1.58	1.45	1.71	1.46	1.35	1.59
Vietnamese	2.42	1.38^*∗*^	1.38	1.28	1.50	1.31	1.22	1.42
Samoan	6.81	3.87^*∗*^	4.29	3.78	4.86	1.66	1.46	1.89
Other Asian/Pacific Islander	3.50	1.99^*∗*^	2.09	1.98	2.20	1.55	1.47	1.63
Mexican	4.28	2.43^*∗*^	2.59	2.48	2.71	1.56	1.49	1.63
Puerto Rican	4.73	2.69^*∗*^	2.90	2.76	3.05	1.67	1.58	1.75
Cuban	4.77	2.71^*∗*^	2.90	2.72	3.08	1.88	1.76	2.00
Central & South American	3.59	2.04^*∗*^	2.13	2.03	2.23	1.64	1.56	1.72
Other Hispanic	4.86	2.76^*∗*^	2.97	2.84	3.11	1.72	1.64	1.80
*Ethnic-immigrant status*								
Non-Hispanic White, US-born	5.84	3.82^*∗*^	4.20	4.41	4.00	2.63	2.77	2.51
Non-Hispanic White, immigrant	3.30	2.16^*∗*^	2.22	2.28	2.16	1.73	1.78	1.68
Non-Hispanic Black, US-born	6.83	4.46^*∗*^	5.06	5.26	4.86	2.72	2.83	2.61
Non-Hispanic Black, immigrant	4.91	3.21^*∗*^	3.35	3.44	3.26	2.34	2.41	2.28
American Indian/Alaska Native	6.77	4.42^*∗*^	5.00	5.10	4.90	2.60	2.66	2.55
Chinese, US-born	3.37	2.20^*∗*^	2.23	2.14	2.33	2.02	1.94	2.11
Chinese, immigrant	1.53	1.00	1.00	Reference		1.00	Reference	
Japanese, US-born	4.75	3.10^*∗*^	3.11	2.81	3.43	2.69	2.44	2.98
Japanese, immigrant	1.92	1.25^*∗*^	1.18	1.07	1.30	1.33	1.22	1.46
Hawaiian	5.62	3.67^*∗*^	4.05	3.46	4.73	2.20	1.88	2.58
Filipino, US-born	6.09	3.98^*∗*^	4.28	4.21	4.34	3.08	3.04	3.13
Filipino, immigrant	5.79	3.78^*∗*^	3.93	3.96	3.90	3.24	3.28	3.22
Asian Indian, US-born	3.74	2.44^*∗*^	2.55	2.44	2.65	1.99	1.91	2.07
Asian Indian, immigrant	3.20	2.09^*∗*^	2.21	2.25	2.18	1.73	1.76	1.71
Korean, US-born	2.91	1.90^*∗*^	1.95	1.76	2.15	1.72	1.56	1.90
Korean, immigrant	2.75	1.80^*∗*^	1.79	1.73	1.84	1.70	1.65	1.75
Vietnamese, US-born	3.77	2.46^*∗*^	2.66	2.48	2.85	2.13	1.99	2.29
Vietnamese, immigrant	2.10	1.37^*∗*^	1.37	1.33	1.41	1.36	1.32	1.39
Samoan	6.81	4.45^*∗*^	5.03	4.68	5.39	2.09	1.95	2.24
Other Asian/Pacific Islander, US-born	4.05	2.65^*∗*^	2.87	2.88	2.86	1.96	1.97	1.95
Other Asian/Pacific Islander, immigrant	3.18	2.08^*∗*^	2.17	2.19	2.15	1.78	1.80	1.76
Mexican, US-born	4.82	3.15^*∗*^	3.50	3.63	3.38	2.00	2.07	1.93
Mexican, immigrant	3.72	2.43^*∗*^	2.58	2.66	2.49	1.81	1.88	1.75
Puerto Rican, mainland US-born	4.69	3.07^*∗*^	3.38	3.45	3.30	2.16	2.20	2.11
Puerto Rican, Puerto Rico-born	5.09	3.33^*∗*^	3.63	3.57	3.70	2.28	2.25	2.33
Cuban, US-born	4.92	3.22^*∗*^	3.51	3.45	3.57	2.21	2.18	2.25
Cuban, immigrant	4.64	3.03^*∗*^	3.26	3.21	3.31	2.39	2.36	2.43
Central & South American, US-born	4.22	2.76^*∗*^	3.02	3.02	3.00	1.95	1.96	1.94
Central & South American, immigrant	3.46	2.26^*∗*^	2.37	2.43	2.31	1.88	1.93	1.83
Other Hispanic, US-born	4.99	3.26^*∗*^	3.63	3.73	3.52	2.13	2.20	2.08
Other Hispanic, immigrant	4.55	2.97^*∗*^	3.17	3.22	3.12	2.35	2.39	2.32
All other ethnic-nativity groups	5.23	3.42^*∗*^	3.71	3.64	3.77	2.65	2.61	2.70

OR = odds ratio; CI = confidence interval. ^*∗*^Statistically significant at *p* < 0.05; ^1^Defined as the ratio of the prevalence for a specific group to that for the reference group. ^2^Adjusted for maternal age only. ^3^Adjusted for maternal age, marital status, nativity, plurality, maternal education, place and region of residence, gestational diabetes, prepregnancy BMI, and gestational weight gain; *Source*. Data derived from the 2014-2015 US National Natality data files.

**Table 5:** Observed prevalence and adjusted odds of *chronic hypertension* among 17 racial/ethnic and 31 ethnic-immigrant groups, United States, 2014-2015 (*N* = 7,966,573).

Ethnicity and immigrant status	Prevalence percent	Prevalence ratio^1^	Model 1^2^			Model 2^3^		
			OR	95% CI		OR	95% CI	
*Race/ethnicity*								
Non-Hispanic White	1.50	3.75^*∗*^	4.87	4.44	5.33	1.59	1.45	1.75
Non-Hispanic Black	3.27	8.18^*∗*^	12.47	11.38	13.67	2.98	2.71	3.27
American Indian/Alaska Native	2.16	5.40^*∗*^	8.60	7.75	9.54	2.04	1.84	2.27
Chinese	0.40	1.00	1.00	Reference		1.00	Reference	
Japanese	0.75	1.88^*∗*^	1.61	1.30	1.98	1.47	1.19	1.82
Filipino	1.86	4.65^*∗*^	4.84	4.34	5.39	3.43	3.08	3.82
Hawaiian	1.33	3.33^*∗*^	4.53	2.97	6.91	1.24	0.81	1.89
Asian Indian	0.72	1.80^*∗*^	2.05	1.84	2.30	1.51	1.35	1.69
Korean	0.83	2.08^*∗*^	1.95	1.68	2.28	1.74	1.49	2.03
Vietnamese	0.51	1.28^*∗*^	1.31	1.12	1.55	1.19	1.01	1.40
Samoan	1.78	4.45^*∗*^	6.55	5.13	8.36	1.48	1.16	1.89
Other Asian/Pacific Islander	0.94	2.35^*∗*^	2.88	2.59	3.20	1.49	1.34	1.66
Mexican	0.89	2.23^*∗*^	3.15	2.87	3.46	1.11	1.01	1.22
Puerto Rican	1.62	4.05^*∗*^	6.15	5.57	6.80	1.67	1.50	1.85
Cuban	1.09	2.73^*∗*^	3.49	3.07	3.98	1.40	1.23	1.60
Central & South American	0.95	2.38^*∗*^	2.95	2.67	3.25	1.33	1.21	1.47
Other Hispanic	1.14	2.85^*∗*^	4.27	3.87	4.70	1.40	1.27	1.54
*Ethnic-immigrant status*								
Non-Hispanic White, US-born	1.55	4.70^*∗*^	2.55	2.73	2.39	1.54	1.65	1.44
Non-Hispanic White, immigrant	0.78	2.36^*∗*^	6.21	6.94	5.59	2.72	3.04	2.44
Non-Hispanic Black, US-born	3.47	10.52^*∗*^	17.53	19.34	15.98	5.14	5.64	4.68
Non-Hispanic Black, immigrant	2.17	6.58^*∗*^	7.10	7.68	6.60	3.01	3.25	2.79
American Indian/Alaska Native	2.16	6.55^*∗*^	10.60	11.27	10.02	3.46	3.66	3.26
Chinese, US-born	0.88	2.67^*∗*^	2.57	2.41	2.74	2.35	2.20	2.52
Chinese, immigrant	0.33	1.00	1.00	Reference		1.00	Reference	
Japanese, US-born	1.70	5.15^*∗*^	4.62	4.05	5.30	4.05	3.53	4.65
Japanese, immigrant	0.40	1.21	0.98	0.81	1.20	1.01	0.82	1.23
Hawaiian	1.33	4.03^*∗*^	5.53	4.09	7.51	2.06	1.52	2.80
Filipino, US-born	1.90	5.76^*∗*^	6.48	6.49	6.50	4.24	4.23	4.25
Filipino, immigrant	1.84	5.58^*∗*^	5.53	5.77	5.32	4.37	4.54	4.20
Asian Indian, US-born	0.78	2.36^*∗*^	2.47	2.28	2.69	1.72	1.58	1.88
Asian Indian, immigrant	0.71	2.15^*∗*^	2.48	2.59	2.39	1.77	1.84	1.70
Korean, US-born	0.91	2.76^*∗*^	2.75	2.37	3.21	2.44	2.09	2.84
Korean, immigrant	0.81	2.45^*∗*^	2.23	2.16	2.32	2.08	2.01	2.16
Vietnamese, US-born	0.78	2.36^*∗*^	2.92	2.53	3.39	2.30	1.98	2.66
Vietnamese, immigrant	0.45	1.36^*∗*^	1.33	1.27	1.41	1.25	1.19	1.32
Samoan	1.78	5.39^*∗*^	8.04	7.17	9.06	2.23	1.98	2.51
Other Asian/Pacific Islander, US-born	1.06	3.21^*∗*^	4.39	4.50	4.29	2.36	2.41	2.30
Other Asian/Pacific Islander, immigrant	0.87	2.64^*∗*^	3.01	3.14	2.92	1.73	1.79	1.67
Mexican, US-born	0.98	2.97^*∗*^	5.02	5.46	4.64	1.89	2.05	1.75
Mexican, immigrant	0.79	2.39^*∗*^	2.97	3.22	2.75	1.23	1.33	1.14
Puerto Rican, mainland US-born	1.57	4.76^*∗*^	7.40	7.90	6.97	2.63	2.80	2.48
Puerto Rican, Puerto Rico-born	1.95	5.91^*∗*^	8.46	8.51	8.45	3.06	3.07	3.06
Cuban, US-born	1.23	3.73^*∗*^	4.98	4.91	5.09	2.17	2.13	2.21
Cuban, immigrant	0.95	2.88^*∗*^	3.60	3.51	3.72	1.78	1.73	1.83
Central & South American, US-born	1.08	3.27^*∗*^	5.01	5.13	4.91	2.16	2.21	2.12
Central & South American, immigrant	0.93	2.82^*∗*^	3.34	3.57	3.13	1.50	1.60	1.41
Other Hispanic, US-born	1.06	3.21^*∗*^	5.32	5.69	5.00	2.05	2.18	1.92
Other Hispanic, immigrant	1.36	4.12^*∗*^	5.06	5.33	4.83	2.23	2.34	2.13
All other ethnic-nativity groups	1.68	5.09^*∗*^	6.74	6.72	6.79	3.04	3.03	3.06

OR = odds ratio; CI = confidence interval. ^*∗*^Statistically significant at *p* < 0.05. ^1^Defined as the ratio of the prevalence for a specific group to that for the reference group. ^2^Adjusted for maternal age only. ^3^Adjusted for maternal age, marital status, nativity, plurality, maternal education, place and region of residence, and prepregnancy BMI. *Source*. Data derived from the 2014-2015 US National Natality data files.
